# Anti-Inflammatory Activity of Two Labdane Enantiomers from *Gymnosperma glutinosum*: An *In Vivo*, *In Vitro*, and *In Silico* Study

**DOI:** 10.3390/ph18040516

**Published:** 2025-04-01

**Authors:** Salud Pérez-Gutiérrez, Nimsi Campos-Xolalpa, Sofía A. Estrada-Barajas, Alan Carrasco-Carballo, Angel Mendoza, Ernesto Sánchez-Mendoza

**Affiliations:** 1 Departamento de Sistemas Biológicos, Universidad Autónoma Metropolitana-Xochimilco, Calzada del Hueso 1100, Coyoacán, Ciudad de México 04960, Mexico; msperez@correo.xoc.uam.mx (S.P.-G.); sofiiabii11@gmail.com (S.A.E.-B.); 2 Secretaria de Ciencia, Humanidades, Tecnología e Innovación (SECIHTI), Departamento de Sistemas Biológicos, Universidad Autónoma Metropolitana-Xochimilco, Calzada del Hueso 1100, Coyoacán, Ciudad de México 04960, Mexico; ncampos@correo.xoc.uam.mx; 3 Secretaria de Ciencia, Humanidades, Tecnología e Innovación (SECIHTI), Laboratorio de Elucidación y Síntesis en Química Orgánica, Instituto de Ciencias, Benemérita Universidad Autónoma de Puebla, Blvd. Capitán Carlos Camacho Espíritu, Puebla 72540, Mexico; alan.carrascoc@correo.buap.mx; 4 Centro de Química, Instituto de Ciencias, Benemérita Universidad Autónoma de Puebla, 18 Sur y Av. San Claudio, Col. San Manuel, Puebla 72570, Mexico; angel.mendoza@correo.buap.mx

**Keywords:** enantiomers, molecular docking, cytokines, cell viability, stabilization of membrane

## Abstract

**Background/Objectives**: Diseases associated with inflammatory processes continue to grow steadily throughout the world. Unfortunately, prolonged use of drugs induces adverse effects ranging from hypersensitivity reactions to damage to the digestive system. These negative effects open the possibility of continuing the search for anti-inflammatory compounds with less toxicity. The aim of this research was to isolate and evaluate the anti-inflammatory activity of a mixture of two enantiomeric labdanes isolated from *Gymnosperma glutinosum* by *in vivo*, *in vitro*, and *in silico* methods. **Methods**: A brief description of the main methods or treatments applied. This can include any relevant preregistration or specimen information. The structure of the labdanes enantiomers was elucidated by X-ray crystallography and spectroscopies methods. The anti-inflammatory effect was evaluated on a mouse model of ear edema induced with 12-O-tetradecanoyl phorbol-13-acetate; the pro-inflammatory mediators, nitric oxide (NO) and interleukin (IL-6), were quantified on macrophages stimulated with lipopolysaccharide, and the interaction between labdanes and diana was studied by molecular docking. **Results**: We identified the chemical structures of two new labdane enantiomers: *a*-gymglu acid and *b*-*ent*-gymglu acid. The enantiomer mixture, named gymglu acid, diminished ear edema at doses of 1 and 2 mg/ear by 36.07% and 41.99%, respectively. A concentration of 155.16 µM of gymglu acid inhibited the production of NO by 78.06% and IL-6 by 71.04%. The *in silico* results suggest two routes by which these labdanes reduce inflammation: partial agonism toward the corticosteroid receptors and inhibition of nitric oxide synthases. **Conclusions**: These results show that the gymglu acid enantiomers have promising anti-inflammatory activity.

## 1. Introduction

The incidence of diseases associated with inflammatory processes continues to grow steadily throughout the global population. Conditions such as rheumatoid arthritis, ulcerative colitis, chronic obstructive pulmonary disease, psoriasis, atherosclerosis, and multiple sclerosis, among others, involve inflammatory processes that can become disabling. The treatment of these diseases is based on the prescription of anti-inflammatory drugs and analgesics. Unfortunately, prolonged use of these compounds induces adverse effects ranging from hypersensitivity reactions to digestive system damage. These negative effects have necessitated the continual search for new compounds with anti-inflammatory activity and fewer adverse effects [1].

Inflammation is a natural defense mechanism used by the body to protect itself against infections and injury. It involves cellular and vascular events, including the activation of monocytes, basophils, eosinophils, and macrophages, as well as the overproduction of some inflammatory mediators, such as nitric oxide (NO), tumor necrosis factor-alpha (TNF-α), interleukin (IL-1β), and IL-6 [2]. Dysregulation of these mediators is associated with different diseases, pain, and hyperalgesia.

According to the World Health Organization (WHO), more than 170 countries have reported the use of traditional medicine as an alternative method for the relief and treatment of different diseases. The use of herbal medicines, extracts, infusions, tonics, and even the direct application of plant material is currently a commonly used alternative treatment in the Mexican population for the relief of various diseases. Therefore, there has been a focused scientific effort to isolate and evaluate the biological activity of different plant metabolites and thereby obtain information that can be applied to the development of new drugs. Furthermore, knowledge of the chemical structure of secondary metabolites allows for the prediction of possible mechanisms of action that explain the observed biological effects with the help of bioinformatics. For example, molecular docking revealed that Z-geranial, a compound identified in *Cymbopogon citratus* essential oil, has greater binding energy than does a commercial M1 muscarinic acetylcholine receptor blocker and blocks the M2 receptor, whereas β-pinene and β-myrcene block the M1, M2, and M4 receptors [3]. These actions may positively affect cardiovascular activity, memory, Alzheimer’s disease, and schizophrenia.

*Gymnosperma glutinosum* (Sprengel) Less. is a plant that has been used in traditional Mexican medicine to combat different diseases. Infusions of this species have been used to treat diarrhea, rheumatism, skin infections, ulcers, and abscesses. *G. glutinosum* belongs to the Asteraceae family; it is commonly known as Tatalencho, Escobilla, Jarilla, and Pegajosa. Scientific studies of this plant have demonstrated the presence of essential oil, flavonoids, and diterpenes [4]. Canales et al. found that methanolic extracts of *G. glutinosum* collected from different regions of Mexico presented antimicrobial and antifungal activity [5]. Moreover, for the first time, they isolated two metabolites from these extracts, namely, (−)-17-hydroxy-neo-clerod-3-en-15-oic acid and 5,7-dihydroxy-3,6,8,2,4,5-hexamethoxyflavone. In addition, compounds with other biological activities have been isolated: (−)-13S,14R,15-trihydroxy-7-oxo-ent-labd-8(9)-ene showed cytotoxic activity against the L5178Y-R lymphoma cell line [6], and (+)-8S,13S,14R,15-ent-labdanetetrol exerted antifungal activity [7]. Other diterpenes have also been isolated, including (+)-ent-labd-7-en-13S,14R,15-triol and (−)-17-hydroxy-neo-clerod-3-en-15-oic acid [8] as well as 2-angeloyl ent-dihydrotucumanoic, which exerts moderate *in vitro* anti-inflammatory and *in vivo* antinociceptive effects [9]. Alonso-Castro et al. reported that 2-angeloyl ent-dihydrotucumanoic has anti-inflammatory, antinociceptive, cytotoxic, and antimicrobial effects [10]. In previous studies conducted by our research group, we demonstrated that the dichloromethane extract of this plant had anti-inflammatory activity in auricular edema in mice induced by TPA [11]. Based on this background, we studied the anti-inflammatory effect of methanolic and dichloromethane extracts of *G. glutinosum*. Furthermore, two enantiomeric labdanes were isolated, and their chemical structures were elucidated. *In vivo*, *in vitro*, and *in silico* methods were used to comprehensively evaluate the anti-inflammatory effects of two labdane enantiomers.

## 2. Results

### 2.1. Isolation and Structural Analysis of Gymglu Acid

Gymglu acid was isolated from the 60:40 hexane-ethyl acetate fraction from the dichloromethane extract, with a yield of 0.067% and a melting point of 189–190 °C. The spectroscopic data, particularly crystallographic studies, confirmed that the white, needle-shaped crystals of gymglu acid are formed by two diterpenoids of the *ent*-labdane type.

#### 2.1.1. Crystallographic Evaluation

We used the CrysAlisPro software (version 1.171.43) to process the X-ray data and to apply numerical absorption correction [12]. We solved the structures with direct methods via the ShelXT program and refined them with full-matrix least-squares based on F^2^ with SHELXL-2019 [13]. We refined the positional and anisotropic atomic displacement parameters for all nonhydrogen atoms. The hydrogen atoms were identified from the Fourier map and included as fixed contributions attached to their parent atoms, with isotropic thermal factors chosen as 1.2 and 1.5 times their respective carrier atoms. We employed the OLEX2 software (version 1.5-alpha) [14] as a graphical interface.

**Figure 1:**
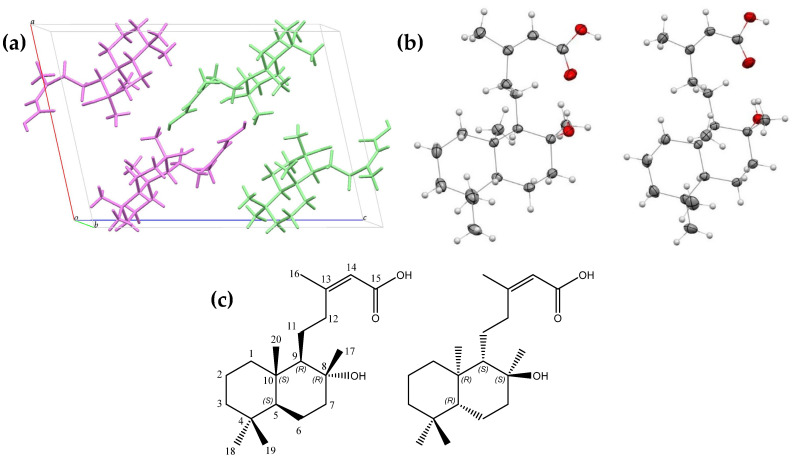
(**a**) Unit cell showing *a*-gymglu acid (violet) and *b-ent*-gymglu acid (green), a, b, and c are the lengths of unit cell edges. (**b**) Molecular structure for each enantiomer showing 30% probability displacement ellipsoids. (**c**) Chemical structure of *a*-gymglu acid and *b-ent*-gymglu acid (IUPAC name in Supplementary Materials).

Crystallographic analysis determined that gymglu acid corresponds to a mixture of two enantiomeric labdanes, with the structural elucidation and stereochemical configuration of the carbons determined as follows: (5S,8R,9R,10S)-8-hydroxy-labda-13(Z)-ene-15-oic acid, which we named *a*-gymglu acid, and (5R,8S,9S,10R)-8-hydroxy-labda-13(Z)-ene-15-oic acid, which we named *b-ent*-gymglu acid (Figure 1a–c). *a*-gymglu acid and *b-ent*-gymglu acid crystallize in a monoclinic system with a P21/c space group, and the asymmetric unit corresponds to C_20_H_34_O_3_. The crystallographic parameters are shown in Table S1. The crystal packing shows two hydrogen bonds: one of them is intramolecular, O1—H1···O2, and the other is intermolecular, O3—H3···O1 (Supplementary Material). The crystallographic data for *a*-gymglu acid and *b-ent*-gymglu acid were deposited in the Cambridge Crystallographic Data Center (CCDC numbers 2364580), and Mercury (Version 2022 3.0) [15] was used to represent crystallographic data.

#### 2.1.2. FT-IR and NMR Evaluation

The FT-IR spectra presented the following peaks: 3408.89 cm^−1^ (OH); 3300.0–2600 cm^−1^ (COOH); 3100 (=C-H); 2923.95, 2889.44, 1458.06, and 1386.37 cm^−1^ (C-H); 1688.88 cm^−1^ (C=O); and 1637.27 cm^−1^ (C=C).

We recorded the ^1^H, ^13^C, gCOSY, TOCSY, gHSQC, and gHMBC NMR spectra in a CDCl_3_ solution at 299 K (NMR spectra are provided in Supplementary Material). We assigned the correlations between protons and carbons based on an analysis of the gCOSY and gHSQC NMR spectra, and we confirmed the structural position of the quaternary carbon based on the gHMBC NMR correlations (Table 1). The complete NMR assignment included the following:

^13^C NMR (151 MHz, CDCl_3_) δ (ppm) C1 39.17, C2 18.45, C3 41.94, C4 33.24, C5 55.97, C6 20.18, C7 42.96, C8 74.06, C9 61.02, C10 38.73, C11 24.23, C12 37.27, C13 161.98, C14 115.86, C15 168.70, C16 25.85, C17 24.21, C18 33.34, C19 21.44, and C20 15.48; ^1^H NMR (600 MHz, CDCl_3_ δ (ppm) H1 1.69 (dd, J = 12.7, 3.5 Hz), H1′ 0.93 (m); H2 1.61 (m), H2′ 1.43 (m), H3 1.37 (dd, J = 13.1, 3.4 Hz), H3′ 1.14 (dd, J = 13.1, 4.2 Hz), H5 0.92 (m), H6 1.64 (m), H6′ 1.25 (m), H7 1.83 (dt, J = 12.5, 3.3 Hz), H7′ 1.44 (m), H9 1.17 (m), H11 1.48 (m), H12 2.95 (td, J = 11.3, 5.0 Hz), H12′ 2.19 (td, J = 11.3, 7.1 Hz), H14 5.68 (d, J = 1.5 Hz), H16 1.91 (d, J = 1.5 Hz), H17 1.17 (s), H18 0.87 (s), H19 0.78, and H20 0.75 (s).

### 2.2. In Vivo and In Vitro Evaluation of Gymglu Acid

The mixture of *a*-gymglu acid and *b-ent*-gymglu acid presented a low yield; thus, separating enantiomers was not possible. Therefore, the *in vivo* and *in vitro* analyses were performed only with the mixture, referred to simply as gymglu acid, for these experiments.

#### 2.2.1. Anti-Inflammatory Activity in a Mouse Ear Edema Induced with TPA

Table 2 presents the anti-inflammatory activity of the dichloromethane and methanol extracts of *G. glutinosum* and indomethacin (positive control) in a mouse ear edema induced with TPA. The dichloromethane extract diminished ear edema by 52.83 ± 5.28%, which was better than that of the methanol extract and comparable to that of indomethacin (49.74 ± 4.25%).

Gymglu acid was isolated from the bioactive extract, and its anti-inflammatory activity was evaluated. We tested two doses (1 and 2 mg) of the gymglu acid on the mouse ear edema induced with TPA (Table 3). The results show that 2 mg/ear dose diminished ear edema by 41.99 ± 3.57%, similar to the effect of indomethacin (50.27 ± 4.46%).

#### 2.2.2. Effect of Gymglu Acid on J774A.1 Macrophages Cell Viability

In accordance with previously described methods, we determined the effect of gymglu acid on the viability of J774A.1 macrophages (Table 4). The results show that the IC_50_ was 390.09 μM. Therefore, in subsequent tests, we used a concentration of 155.04 μM, which did not affect the 70% cell viability.

#### 2.2.3. Measurement of Proinflammatory Mediator Levels

**Figure 2:**
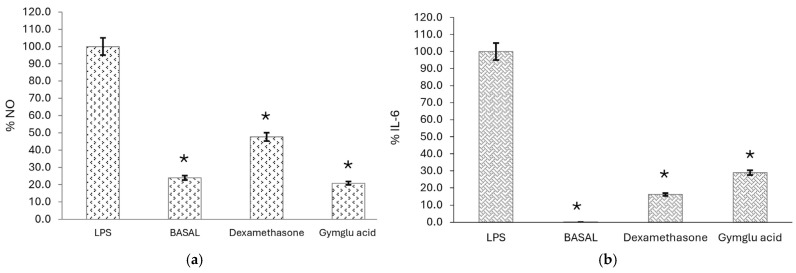
The effect of gymglu acid (155.04 µM) and dexamethasone (12.74 µM) on the release of (**a**) NO and (**b**) IL-6 from J774A.1 macrophages stimulated with LPS. The graph represents the mean ± S.E.M. of three independent experiments (n = 4). * *p* < 0.05 compared with the LPS group.

As mentioned above, we stimulated J774A.1 macrophages with LPS to induce the release of pro-inflammatory mediators such as NO and IL-6. Findings revealed that gymglu acid at a concentration of 155.04 µM inhibited the production of NO by 79.18%, a stronger effect compared with dexamethasone (52.32%) (*p* < 0.05). Moreover, gymglu acid at the same concentration inhibited the production of IL-6 (71.04%), while the reference compound did the same at 83.8% (*p* < 0.05) (Figure 2).

#### 2.2.4. The Membrane Protective Effect of Gymglu Acid

The membrane protective effect of gymglu acid was evaluated, as presented in Table 5; increasing the gymglu acid concentration increased the percentage of erythrocyte membrane protection. This behavior is similar to that of diclofenac. However, the IC_50_ of diclofenac (38.81 µg/mL) was lower than gymglu acid (94.47 µg/mL).

### 2.3. In Silico Results

**Figure 3:**
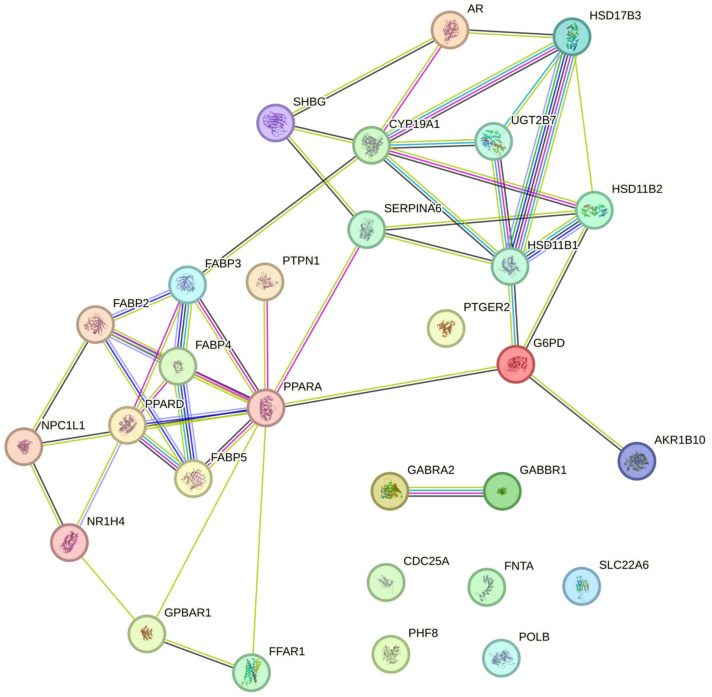
The relationship between the isolated molecules (*a*-gymglu acid and *b*-*ent*-gymglu acid) and interacting proteins.

As the *a*-gymglu acid and *b-ent*-gymglu acid may interact differently with the organism during the inflammatory process, bioinformatics studies were realized with the enantiomeric structures. Based on the chemical structures, we searched for the molecular targets underlying the anti-inflammatory and antioxidant activities of *a*-gymglu acid and *b-ent*-gymglu acid. We performed structural similarity analysis via SwissTargetPrediction in combination with STRING relationship analysis (Figure 3). The second pathway focuses on oxidative stress via PPARA and the downstream response was triggered by this regulation (Figure 3).

We assessed the MR, GR, HSD11B1, PPARA, nNOS, iNOS, and eNOS as molecular targets by molecular docking and MM-GBSA. The results showed the regulation via NOSs based on molecular docking calculations. The results presented in Table 6 indicate that the *b-ent*-gymglu acid has even greater coupling than the reference for the PPARA pathway, suggesting it may be an antagonist of this pathway.

**Figure 4:**
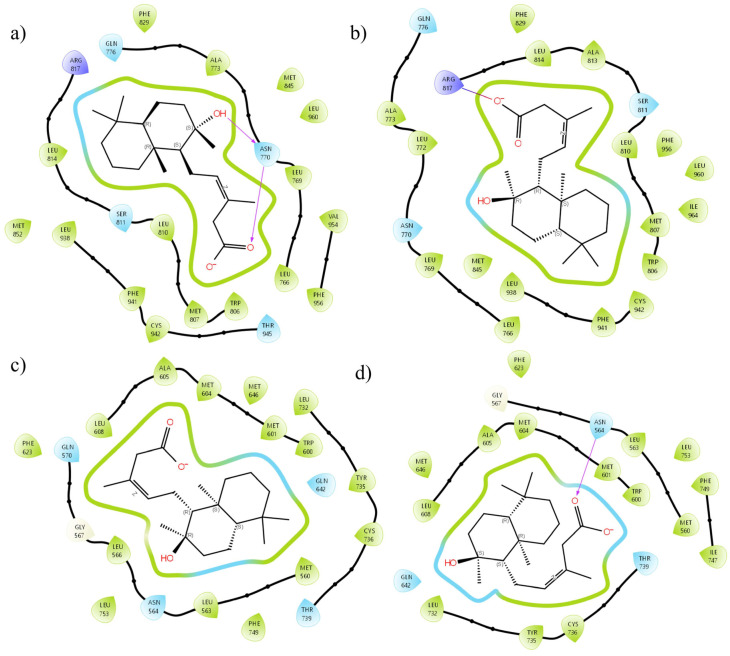
A two-dimensional interaction diagram of (**a**) *b*-*ent*-gymglu acid with the MR, (**b**) *a*-gymglu acid with the MR, (**c**) *b*-*ent*-gymglu acid with the GR, and (**d**) *a*-gymglu acid with the GR.

In contrast to the corticosteroid route, we observed a partial agonism for the MR and the GR, indicating a direct relationship between the dose and the anti-inflammatory effect. Figure 4 presents the differences in the interactions between the enantiomers and the sites of action. For the MR, the carboxylate of *b-ent*-gymglu acid interacts via Arg817 (Figure 4b), whereas for *a*-gymglu acid, it interacts via Asn770 (Figure 4a), indicating that the three-dimensional arrangement of the enantiomers plays a very important role in the way these molecules couple with their receptors. For example, when coupled with GR, only *a*-gymglu acid hydrogen bonds with Asn564 (Figure 4d); however, both structures maintain an amphipathic environment, characteristic of partial activation of this receptor. In addition, regarding the regulation of corticosteroid production, only *a*-gymglu acid has sufficient energy to overcome cortisol, leading to inhibition of the cortisol to cortisone pathway and resulting in increased cortisol levels. Nevertheless, this action deregulates the system, so the dose is important for regulating the pathway and thus, the cortisol levels.

Finally, when analyzing the effects on NOSs, we found a preference for iNOS, followed by eNOS and nNOS (Table 4). These findings indicate a certain specificity for systemic regulation prior to regulation of the central nervous system. This phenomenon explains the decrease in NO levels caused by both compounds. To summarize, the enantiomeric mixture has multitarget activity, starting from partial agonism of the GR and MR, as well as a downregulation of oxidative stress via selective inhibition of NOSs.

## 3. Discussion

In the present study, for the first time, we obtained a white crystalline solid from the dichloromethane extract of *G. glutinosum*, which we identified as an enantiomeric mixture of labdanes. Many reports suggest that these diterpenoids have a wide variety of bioactivities, including antibacterial, cytotoxic, and anti-inflammatory effects, as well as hepatoprotective and neuroprotective effects. In this work, we found that gymglu acid exhibited anti-inflammatory activity because it inhibited TPA-induced ear edema in mice. TPA leads to edema, erythema, and infiltration of neutrophils and leukocytes and activates protein kinase C [16] and phospholipase A2 [17]. These results suggest that gymglu acid can inhibit the activation of some of these enzymes and inflammatory cells.

NO is involved in physiological functions such as vasodilation and neuronal transmission; it is produced by monocytes, macrophages, and neutrophils as part of the immune response [18]. NO is produced by constitutively expressed NOS isoforms (eNOS and nNOS) as well as by iNOS, which promote the formation of reactive nitrogen species that are involved with tissue damage. NO overexpression promotes the release of inflammatory cytokines, such as IL-1β, TNF-α, and IL-6, among others [19]. IL-6 has pleiotropic activity and plays an important role in the acquired immune response by stimulating the production of antibodies and the development of effector T cells; however, continuous unregulated production of IL-6 leads to the development of different diseases such as those related to inflammation [20]. Therefore, compounds that inhibit the production of NO and IL-6 could be used to treat different inflammatory problems. Our results demonstrated that gymglu acid reduced NO and IL-6 production in LPS-stimulated macrophages. These conclusions are supported by previously published studies that have shown that some diterpene labdanes have anti-inflammatory activity via the inhibition of NF-κB activity, modulation of arachidonic acid metabolism, and reduction of NO production [21].

During the inflammatory response, neutrophils release their lysosomal components, such as proteases and bactericidal enzymes, further increasing inflammation. Lysosomal membrane components are analogous to human erythrocyte membranes [22]. We found that gymglu acid protected the human erythrocyte membrane from hemolysis, with protection increased as the gymglu acid concentration increased.

The *in silico* results suggest two routes by which *a*-gymglu acid and *b-ent*-gymglu acid exert their effects, and biological results are the result of the sum of both. The first mechanism, with a lesser impact, involves partial agonism of the corticosteroid receptors, which can partially explain the ability of *a*-gymglu acid to reduce inflammation. Indeed, GR agonism has been reported to have anti-inflammatory effects. Moreover, partial agonism of the GR and the MR may underlie the positive effects of both enantiomers on hemolysis [23]. The second route is the inhibition of NOSs: *b-ent*-gymglu acid predominately inhibits iNOS, which is directly correlated with its ability to reduce NO production in LPS-stimulated macrophages. However, in further studies, the activity of gymglu on iNOS and GR will be determined.

In summary, the enantiomers studied presented inhibitory inflammatory effects based on the *in silico*, *in vitro*, and *in vivo* results. Therefore, the anti-inflammatory effects of gymglu acid make it a very relevant active ingredient that could be promising to treat acute and chronic inflammation. Therefore, this study suggests that gymglu acid could have the potential to be an alternative anti-inflammatory agent.

## 4. Materials and Methods

### 4.1. Materials

Fetal bovine serum (FBS), Dulbecco’s Modified Eagle’s Medium (DMEM), penicillin-streptomycin antibiotics, indomethacin, 3-(4,5-dimethylthiazol-2-yl)-2,5-diphenyl tetrazolium bromide (MTT), the Griess reagent, dimethyl sulfoxide (DMSO), lipopolysaccharide (LPS), polyvinylpyrrolidone (PVP), and 12-O-tetradecanoylphorbol-13-acetate (TPA) were purchased from Sigma Aldrich (St. Louis, MO, USA). The enzyme-linked immunosorbent assay (ELISA) kits were obtained from TONBO Bioscience (San Diego, CA, USA).

### 4.2. Plant Material

The plant was gathered through ecological pruning, and aerial parts of *G. glutinosum* were collected on highway 70, San Luis Potosí—Rio Verde, in August 2018. The plant was identified by M.Sc. Gabriel Flores Franco of the Universidad Autónoma del Estado de Morelos (UAEM). A voucher specimen (HUMO35267) was deposited in the herbarium (HUMO) of the UAEM.

### 4.3. Obtaining Extracts

The aerial parts of *G. glutinosum* were dried in the shade at room temperature. Subsequently, 300 g of previously ground plant was macerated in 2 L of dichloromethane or methanol for 8 days. Then, the mixture was filtered, and the solvent was removed on a rotatory evaporator under reduced pressure. The anti-inflammatory activity of the extracts was evaluated in a mouse ear edema model induced with TPA.

### 4.4. Isolation of a White Crystalline Solid: Gymglu Acid

The bioactive extract was separated by an open-column chromatography (column height 70 cm; column diameter 3 cm) packed with silica gel (Macherey-Nagel 60, 70–230 mesh ASTM). The mobile phase comprises hexane with increasing amounts of ethyl acetate to increase polarity; 100 mL of each fraction was collected. A white crystalline solid, named gymglu acid, was obtained from the 60:40 hexane–ethyl acetate fraction. The anti-inflammatory activity of this compound was subsequently evaluated in a mouse ear edema model induced with TPA.

### 4.5. Structural Analysis of the Solid Crystalline

Gymglu acid was analyzed via different spectroscopic techniques: Fourier transform infrared (FTIR) spectra were obtained via a Spectrum Two FT-IR spectrometer (Perkin Elmer, Waltham, MA, USA) with attenuated total reflectance (ATR). Nuclear Magnetic Resonance (NMR) spectra were recorded in a deuterated chloroform (CDCl_3_) solution at 299 K on a DD2 600 spectrometer (Agilent, Palo Alto, CA, USA). The ^1^H and ^13^C NMR chemical shifts were reported relative to those of TMS and CDCl_3_, respectively. From a previously reported fraction, suitable crystals of gymglu acid were obtained via slow evaporation. For X-ray diffraction, data were collected with an Oxford Diffraction Gemini-Atlas diffractometer equipped with a charge-coupled device area detector and graphite monochromate Mo-Kα radiation (λ = 0.71073 Å).

### 4.6. Animals

Male CD1 mice weighing 25–30 g were supplied by the Universidad Autónoma Metropolitana-Xochimilco (UAM-X) bioterium. The animals had free access to water and food and were kept at 20–22 °C under a 12 h photoperiod. The study was conducted according to the Official Mexican Standard (NOM-062-ZOO-1999) [24]. The protocol (number 140) was approved by the Research Bioethics Committee of UAM-X.

### 4.7. Anti-Inflammatory Activity in Mouse Ear Edema Induced with TPA

Groups were formed with eight mice each and treated as follows: a solution of 2.5 µg of TPA in 20 µL of acetone was topically applied to the right ear of each mouse. After 30 min, one of the following treatments was applied on the right ear by group: 20 µL of vehicle (negative control), 2 mg of indomethacin (positive control), 2 mg of dichloromethane or methanol extract, or 1 or 2 mg of gymglu acid (test groups). All extracts or compounds were dissolved in 20 µL of the vehicle (dichloromethane, acetone or methanol), and in the left ear to all mice, 20 µL of the vehicle was administered. Six hours after the administration of TPA, the mice were euthanized in a CO_2_ chamber, and a central 6 mm portion was obtained from both ears of the mice. These portions were weighed to calculate the percentage of edema inhibition according to the following equation [25]:

$$\%\;inhibition=\left(\frac{\left(Wt-Wnt\right)control-\left(Wt-Wnt\right)treated}{\left(Wt-Wnt\right)control}\right)\times 100$$

where Wt is the weight of the treated ear, and Wnt is the weight of the non-treated ear.

### 4.8. Cell Viability Assay

J774A.1 cells murine macrophage were cultured in 96-well plates (5 × 10^3^ cells per well) with DMEM containing 10% FBS, HEPES/NaOH (20 mM) at pH 7.4, NaHCO_3_ (2 mM), antibiotics including 100 μg/mL streptomycin and 100 U/mL penicillin at 37 °C, and 5% CO_2_. Twenty-four hours later, the cells were treated with 3.10, 15.50, 31.03, 77.52, 155.16, 310.32, or 620.64 µM gymglu acid in phosphate-buffered saline (PBS). Untreated cells served as a negative control. Forty-eight hours after treatment, 10 μL MTT (5 mg/mL) was added, and the plates were incubated for 4 h. Next, the medium was removed, and the formazan crystals were dissolved in 100 µL DMSO. The absorbance was measured at 540 nm via an ELISA plate reader (spectrophotometer, Bio-Rad Laboratories Inc., Hercules, CA, USA). The half-maximal inhibitory concentration (IC_50_) was calculated via linear regression analysis.

### 4.9. Determination of NO and IL-6 Levels

Initially, 5 × 10^5^ J774A.1 macrophages were seeded per well in 6-well plates; they were then stimulated with 5 µg/mL LPS. Two hours after treatment with 155.16 µM gymglu acid, 12.74 µM dexamethasone, or PBS, the cells were stimulated with LPS. After 24 h, the supernatants were collected for NO and IL-6 quantification. For NO analysis, 100 µL Griess reagent and 100 µL supernatant were mixed, the mixture was incubated for 30 min at 37 °C, and the absorbance was read at 540 nm with an ELISA plate reader (Bio-Rad spectrophotometer). The percentage of NO production was calculated based on the group treated with only LPS as a reference [26]. To quantify IL-6, a portion of the supernatant was analyzed via a commercial ELISA following the manufacturer’s instructions (Promega, Madison, WI, USA). The absorbance was read at 405 nm via a spectrophotometer.

### 4.10. Membrane Stabilization Assay

Blood samples were collected by the physician of the UAM-X medical service from a healthy volunteer under aseptic conditions. The volunteer signed a letter of consent and declared had not consumed nonsteroidal anti-inflammatory drugs, steroidal anti-inflammatory drugs, or contraceptives 2 weeks prior to collection. The blood samples (10 mL) were washed with an equal volume of Alsever’s solution (2% dextrose, 0.8% sodium citrate, 0.05% citric acid, and 0.42% sodium chloride in water) and centrifuged at 3000 rpm for 10 min. Subsequently, a 10% (*v*/*v*) suspension of erythrocytes with isosaline solution (HRBC) was prepared.

Three groups (n = 4) were established; each tube was prepared with 150 µL of HRBC and 550 µL of hyposaline solution (0.036%), and 300 µL of the sample to be evaluated was added. Phosphate buffer was added to the negative control group; diclofenac was added to the positive control group; and gymglu acid was added to the test group at concentrations of 6.25, 12.5, 25, 50, 100, and 200 µg/mL.

All the samples were incubated at 37 °C for 30 min; subsequently, the samples were centrifuged at 3500 rpm for 5 min, and then 100 µL of the supernatant was added to a 96-well plate. The absorbances were read at 540 nm via a spectrophotometer. The erythrocyte membrane protection percentages were calculated via the following formula [27]:

$$\%\;Hemolysis=\frac{absorbance\;of\;test\;sample}{Absorbance\;of\;control}\times 100$$

### 4.11. Statistical Analysis

The results are expressed as the mean ± standard error of the mean (S.E.M.). Statistical analyses were performed with analysis of variance (ANOVA) followed by Tukey’s post hoc test, using the statistical program iner-STAT20-a v1.3. In the figures and tables, asterisks (*) indicate statistical significance: * *p* < 0.05.

### 4.12. Bioinformatics

Based on the structures of the crystals and respecting stereochemistry, the simplified molecular input line entry system (SMILES) was constructed for structural similarity analysis in SwissTargetPrediction (http://www.swisstargetprediction.ch/) [28], together with the main targets previously obtained through STRING [29].

#### 4.12.1. Protein Preparation

The proteins of interest are the mineralocorticoid receptor (MR, 3VHU), the glucocorticoid receptor (GR, 4CSJ), the 11β-hydroxysteroid dehydrogenase type 1 (HSD11B1, 2RBE), the peroxisome proliferator-activated receptor alpha (PPARA, 6GHK), neural nitric oxide synthase (nNOS, 4EUX), inducible nitric oxide synthase (iNOS, 4NOS), and endothelial nitric oxide synthase (eNOS, 3NOS) were prepared in Schrödinger’s Protein Preparation Wizard (Version 2023-2) [30], the protonation and tautomeric states of Asp, Glu, Arg, Lys, and His were adjusted to a pH of 7.4. Water molecules inside a sphere of 5 Å in the active site were removed. The orientation of the hydrogen bridge bonds was adjusted around the active site using PROPKA at a pH of 7.4, and water molecules had one or two hydrogen bridges removed. Finally, OPLS4 was used to minimize the complete protein at 0.3 Å root mean square deviation according to the previously reported protocols [31].

#### 4.12.2. Ligand Preparation

The ligation of the crystallized structure was minimized, which led to dissolution conditions and a physiological pH of 7.4 in Macromodel and LigPrep (Schrödinger, Version 2022-1). Water was used as a solvent for minimization, along with the PGRP minimization model with OPLS4 force fields. For LigPrep with OPLS4 force field, all possible protonated centers and ionization states were calculated for scaffolding using an Ionizer at pH 7.4. Stereoisomers were retained according to their original crystal structures, which were limited to 32 isomers for each ligand. Tautomeric states were generated for each group. Conformers with lower energy for each ligand were selected [32].

#### 4.12.3. Molecular Docking

The docking study was carried out in Glide, allowing flexibility at the interaction site and with XP precision. Each grid box was built based on co-crystallized reference ligands or inhibitors; the softening of the nonpolar parts of the receptors was carried out by scaling the van der Waals radii by a 0.08 factor. Atoms were considered nonpolar if they were determined that their absolute partial atomic charge was <0.25. In flexibility, additional ligand rotations were allowed for the hydroxyl groups in Ser, Thr, and Tyr, and the thiol group in Cys residues. Also, the lowest energy binding position of each ligand was maintained. First, docking with the reference molecules of the respective protein targets was performed to validate the docking protocol and select the best coupling by Schrödinger software (2022-1) according to a previously established protocol [32].

#### 4.12.4. MMGBSA Calculations

The binding energy of the selected gymglu acid and references were analyzed using Molecular Mechanics with MM-GBSA of prime Schrödinger molecule [33,34,35]. The solvation model was set to VSGB (Variable Dielectric Surface Generalized Born) and the force field to OPLS4. Protein residues were delimited 12 Å from the ligand for energy minimization. The binding free energy was calculated by:

ΔG_Binding_ = G_Complex_ − (G_Protein_ − G_ligand_)

where ΔG_Binding_ = binding free energy, G_Complex_ = free energy of the complex, G_Protein_ = free energy of the target protein and G_ligand_= free energy of the ligand.

## Figures and Tables

**Table 1:** NMR Correlations.

Carbon	HSQC Correlations	HMBC Correlations
1	39.17: 1.96, 0.93	C1, H20
2	18.45: 1.60, 1.43	C2, H3′
3	41.94: 1.37, 1.14	C3, H18; C3, H19
4	33.24	C4, H18; C4, H19
5	55.97: 0.92	C5, H20; C5, H19; C5, H18
6	20.18: 1.64, 1.25	C6, H5; C6, H7′
7	42.96: 1.83, 1.44	C7, H5; C7, H17
8	74.06	C8, H17; C8, H9; C8, H7; C8, H7′; C8, H11
9	61.02: 1.17	C9, H12; C9, H12′; C9, H7; C9, H7′; C9, H17; C9, H20; C9, H5; C9, H11
10	38.73	C10, H5; C10, H20
11	24.23: 1.48	C11, H12; C11, H12′
12	37.27: 2.95, 2.19	C12, H14; C12, H16; C12, H11
13	161.98	C13, H12; C13, H12′; C13, H19; C13, H11; C13, H14
14	115.86: 5.68	C14, H12; C14, H12′; C14, H16
15	168.70	C15, H14
16	25.85: 1.91	C16, H14; C16, H12; C16, H12′
17	24.21: 1.17	C17, H12; C17, H12′; C17, H9; C17, H7; C17, H7′
18	33.34: 0.87	C18, H19; C19, H5; C18, H3′
19	21.44: 0.78	C19, H18; C19, H5; C19, H3′
20	15.48: 0.75	C20, H5; C20, H9

**Table 2:** The anti-inflammatory activity of the *G. glutinosum* extracts and indomethacin on a mouse ear edema induced with 12-O-tetradecanoyl phorbol-13-acetate.

Group	Percent Decrease in Inflammation	Weight Differences (mg)
Methanol extract (2 mg/ear)	41.96 ± 6.5 *	6.14 ± 0.69 ^#^
Dichloromethane extract (2 mg/ear)	52.83 ± 5.28	4.99 ± 0.56 ^#^
Indomethacin (2 mg/ear)	49.74 ± 4.25	5.31 ± 0.48 ^#^
Negative	------	10.58 ± 0.41

The values are the mean ± S.E.M. (n = 8). *p* < 0.05 versus the * Indomethacin group and ^#^ Negative group.

**Table 3:** The anti-inflammatory activity of gymglu acid on a mouse model of ear edema induced with 12-O-tetradecanoyl phorbol-13-acetate.

Group	Percent Decrease in Inflammation	Weight Differences (mg)
Gymglu acid (1 mg/ear)	36.07 ± 0.84 *	7.57 ± 0.19 ^#^
Gymglu acid (2 mg/ear)	41.99 ± 3.57 *	6.7 ± 0.44 ^#^
Indomethacin (2 mg/ear)	50.27 ± 4.46	5.7 ± 0.52 ^#^
Negative	------	11.55 ± 0.36

The values are the mean ± S.E.M. (n = 8). *p* < 0.05 versus the * Indomethacin and ^#^ Negative group.

**Table 4:** Percentage of the viability of the J774A.1 macrophages treated with gymglu acid.

µM	% Cell Viability
0	100 ± 3.32
3.11	92.93 ± 2.86
15.53	83.79 ± 1.93
31.06	81.92 ± 1.75
77.64	73.33 ± 2.13
155.28	74.27 ± 1.86
232.92	71.24 ± 1.82
310.56	57.03 ± 2.05

The values are the mean ± S.E.M.

**Table 5:** Percentage of the hemolysis of the erythrocyte membrane treated with diclofenac and gymglu acid.

Concentration (μg/mL)	% Erythrocyte Membrane Protection	
	Diclofenac	Gymglu Acid
200	63.04 ± 3.98	56.43 ± 0.95
100	61.30 ± 0.54	52.46 ± 0.59
50	51.15 ± 3.05	30.24 ± 0.48
25	45.96 ± 4.81	17.69 ± 1.69
12.5	31.39 ± 5.17	8.57 ± 1.35
0	100.00 ± 0.59	100.00 ± 0.58
IC_50_ (µg/mL)	38.806	94.47

The values are the mean ± S.E.M. (n = 8).

**Table 6:** Docking scores for *a*-gymglu acid and *b*-*ent*-gymglu acid with select proteins of interest.

Protein	*a*-Gymglu Acid	*b-ent*-Gymglu Acid	Reference
PPARA	NI/NI	−6.229/−55.19	−4.287/−43.56 (thiazolidinediones)
MR	−7.245/−83.98	−6.016/−67.30	−9.324/−93.64 (dexamethasone)
GR	−7.867/−19.98	−7.019/−17.37	−10.258/−31.02 (dexamethasone)
HSD11B1	−6.095/−36.87	−5.064/−39.36	−6.038/−37.26 (glycyrrhizic acid)
iNOS	−4.025/−9.96	−4.318/−12.38	−3.876/−11.38 (L-NMMA)
eNOS	−4.128/−10.12	−3.975/−11.52	−3.957/−11.45 (L-NMMA)
nNOS	−3.017/−9.35	−3.358/−10.98	−3.797/−11.92 (L-NMMA)

NI—no interaction encounter.

## Data Availability

The research data are contained within the article and Supplementary Materials.

## Supplementary Materials

The following supporting information can be downloaded at: https://www.mdpi.com/article/10.3390/ph18040516/s1, Table S1. Crystal data and structure refinement for *a*-gymglu acid and *b-ent*-gymglu acid; Table S2. Hydrogen Bonds for *a*-gymglu acid and *b-ent*-gymglu acid; Chemical structure and IUPAC name for *a*-gymglu acid and *b-ent*-gymglu acid; IR spectrum and NMR spectra for gymglu acid.

## Abbreviations

The following abbreviations are used in this manuscript:

ATR	Attenuated total reflectance
CDCl_3_	Deuterated chloroform
DMEM	Dulbecco’s Modified Eagle’s Medium
DMSO	Dimethyl sulfoxide
ELISA	Enzyme-linked immunosorbent assay
eNOS, 3NOS	Endothelial nitric oxide synthase
FBS	Fetal bovine serum
FTIR	Fourier-transform infrared
GR, 4CSJ	Glucocorticoid receptor
HRBC	Suspension of erythrocytes with isosaline solution.
HSD11B, 2RBE	Hydroxysteroid 11-beta dehydrogenase 1
IL-1β	Interleukin one beta
IL-6	Interleukin six
IND	Indomethacin
iNOS, 4NOS	Inducible nitric oxide synthase
J774A.1	Cells murine macrophage
MR	Mineralocorticoid receptor
MTT	3-(4,5-dimethylthiazol-2-yl)-2,5-diphenyl tetrazolium bromide
NMR	Nuclear magnetic resonance
nNOS, 4EUX	Neuronal nitric oxide synthase
NO	Nitric oxide
LPS	Lipopolysaccharide
PBS	Phosphate-buffered saline
PPARA, 6GHK	Peroxisome proliferator-activated receptor Alpha
PVP	Polyvinylpyrrolidone
SMILES	Simplified Molecular Input Line Entry System
TNF-α	Tumor Necrosis Factor Alpha
TPA	12-O-tetradecanoyl phorbol-13-acetate
3VHU	Mineralocorticoid receptor ligand-binding domain with spironolactone
