# Supplementary material for: Anti-Inflammatory Activity of Two Labdane Enantiomers from Gymnosperma glutinosum: An In Vivo, In Vitro, and In Silico Study
Source: Pharmaceuticals (Basel). 2025 Apr 1;18(4):516. doi: 10.3390/ph18040516 (PMC12030748; doi:10.3390/ph18040516)
Supplement: Supplementary file 1 [file pharmaceuticals-18-00516-s001.zip › pharmaceuticals-3527372-supplementary.pdf]

Table S1. Crystal data and structure refinement for *a*-gymglu acid and *b-ent*-gymglu acid.

Empirical formula	$C_{20}H_{34}O_3$
Formula weight	322.47
Temperature/K	293(2)
Crystal system	monoclinic
Space group	$P2_1/c$
a/Å	13.6127(5)
b/Å	7.2209(3)
c/Å	19.8271(7)
α/°	90
β/°	103.318(4)
γ/°	90
Volume/Å^3	1896.51(13)
Z	4
ρ_{calc}g/cm^3	1.129
μ/mm^{-1}	0.074
F(000)	712.0
Crystal size/mm^3	0.53 × 0.48 × 0.19
Radiation	Mo *Kα* (λ = 0.71073)
2Θ range for data collection/°	6.024 to 61.01
Index ranges	-19 ≤ h ≤ 19, -10 ≤ k ≤ 10, -28 ≤ l ≤ 28
Reflections collected	53346
Independent reflections	5782 [R_{int} = 0.0448, R_{sigma} = 0.0238]
Data/restraints/parameters	5782/0/215
Goodness-of-fit on F^2	1.041
Final R indexes [I>=2σ (I)]	R_1 = 0.0475, wR_2 = 0.1264
Final R indexes [all data]	R_1 = 0.0678, wR_2 = 0.1431
Largest diff. peak/hole / e Å^{-3}	0.23/-0.17

Table S2. Hydrogen Bonds for *a*-gymglu acid and *b-ent*-gymglu acid.

D	H	A	d(D-H)/Å	d(H-A)/Å	d(D-A)/Å	D-H-A/°
O1	H1	O2	0.82	2.03	2.7953(13)	156.2
O3	H3	O1[1]	0.82	1.88	2.6960(13)	170.0

[1]1-X,-Y,1-Z

a-gymglu acid

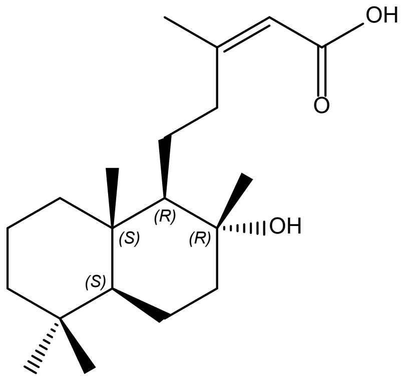

(Z)-5-((1R,2R,4aS,8aS)-2-hydroxy-2,5,5,8a-tetramethyldecahydronaphthalen-1-yl)-3-methylpent-2-enoic acid

b-*ent*-gymglu acid

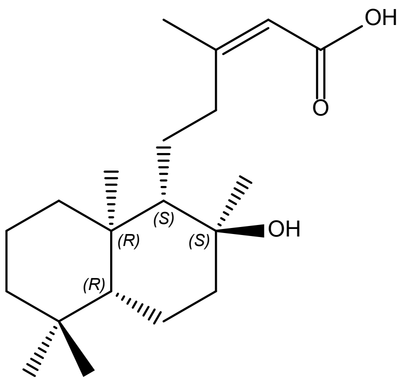

(Z)-5-((1S,2S,4aR,8aR)-2-hydroxy-2,5,5,8a-tetramethyldecahydronaphthalen-1-yl)-3-methylpent-2-enoic acid

Chemical Formula: $C_{20}H_{34}O_3$

Exact Mass: 322.25

Molecular Weight: 322.49

Gymglu acid

Date: Wednesday, June 28, 2023

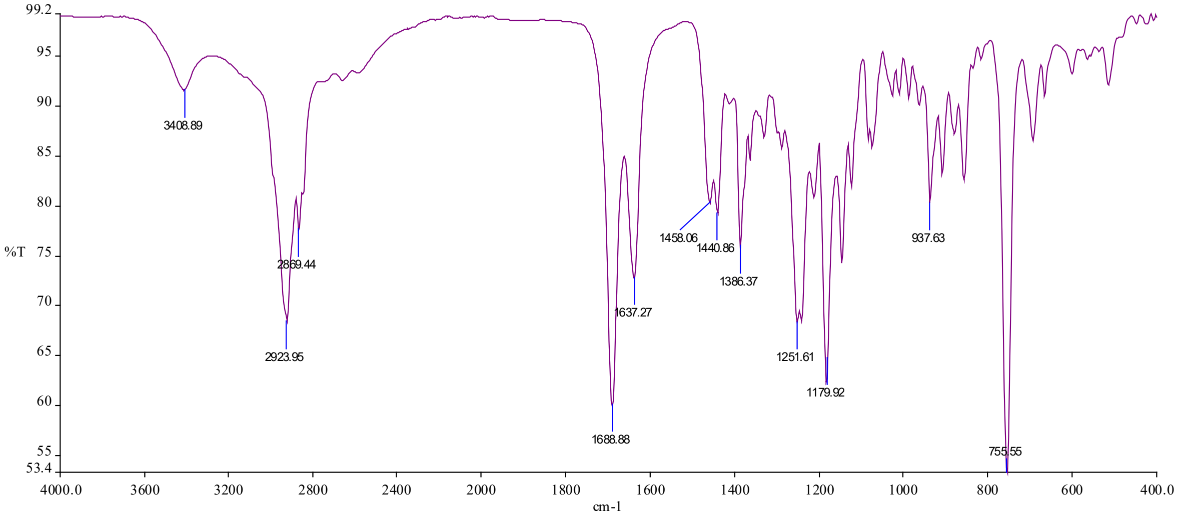

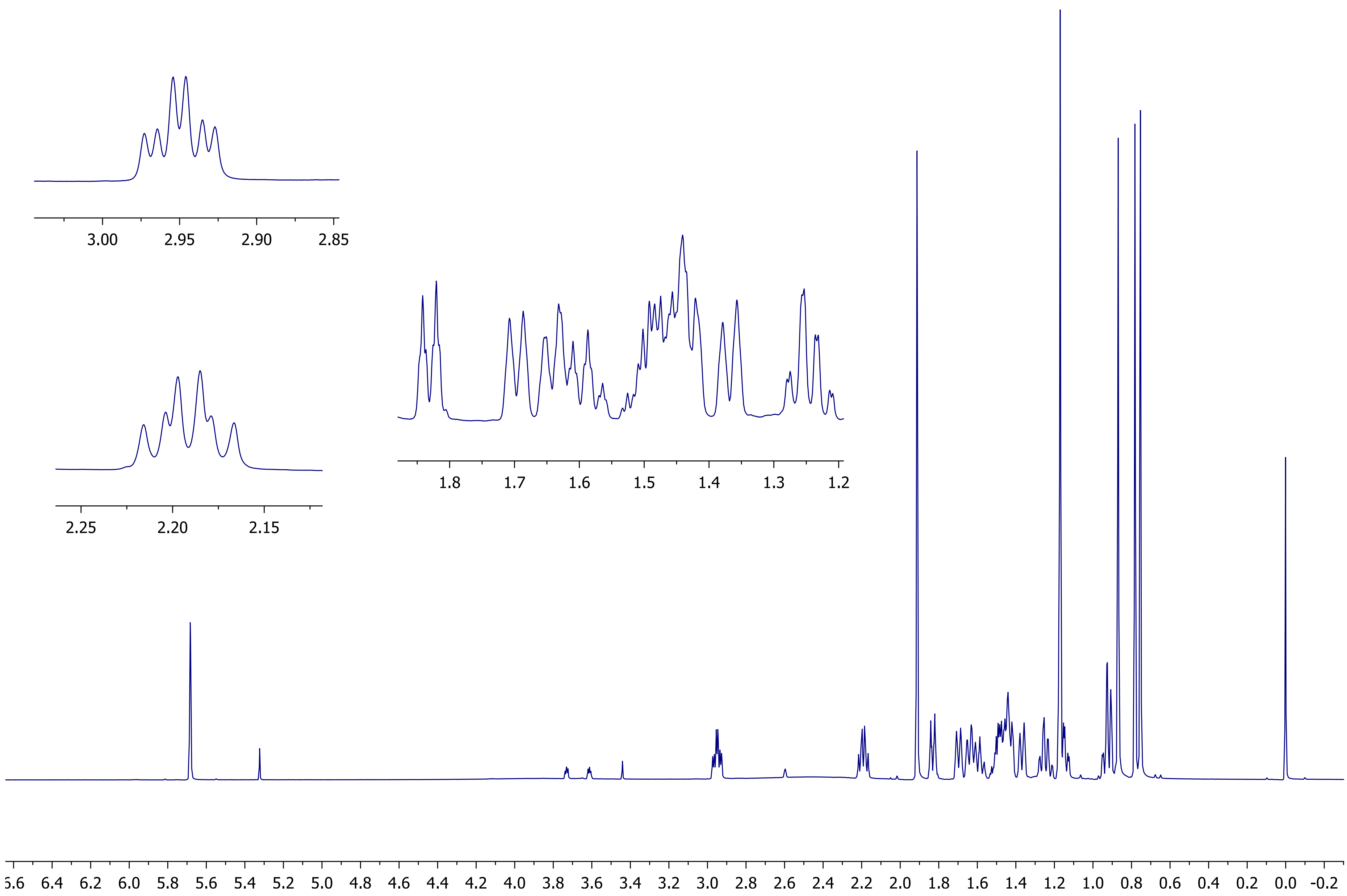

3.00
2.95
2.90
2.85
2.25
2.20
2.15
1.8
1.7
1.6
1.5
1.4
1.3
1.2
6.6
6.4
6.2
6.0
5.8
5.6
5.4
5.2
5.0
4.8
4.6
4.4
4.2
4.0
3.8
3.6
3.4
3.2
3.0
2.8
2.6
2.4
2.2
2.0
1.8
1.6
1.4
1.2
1.0
0.8
0.6
0.4
0.2
0.0
-0.2

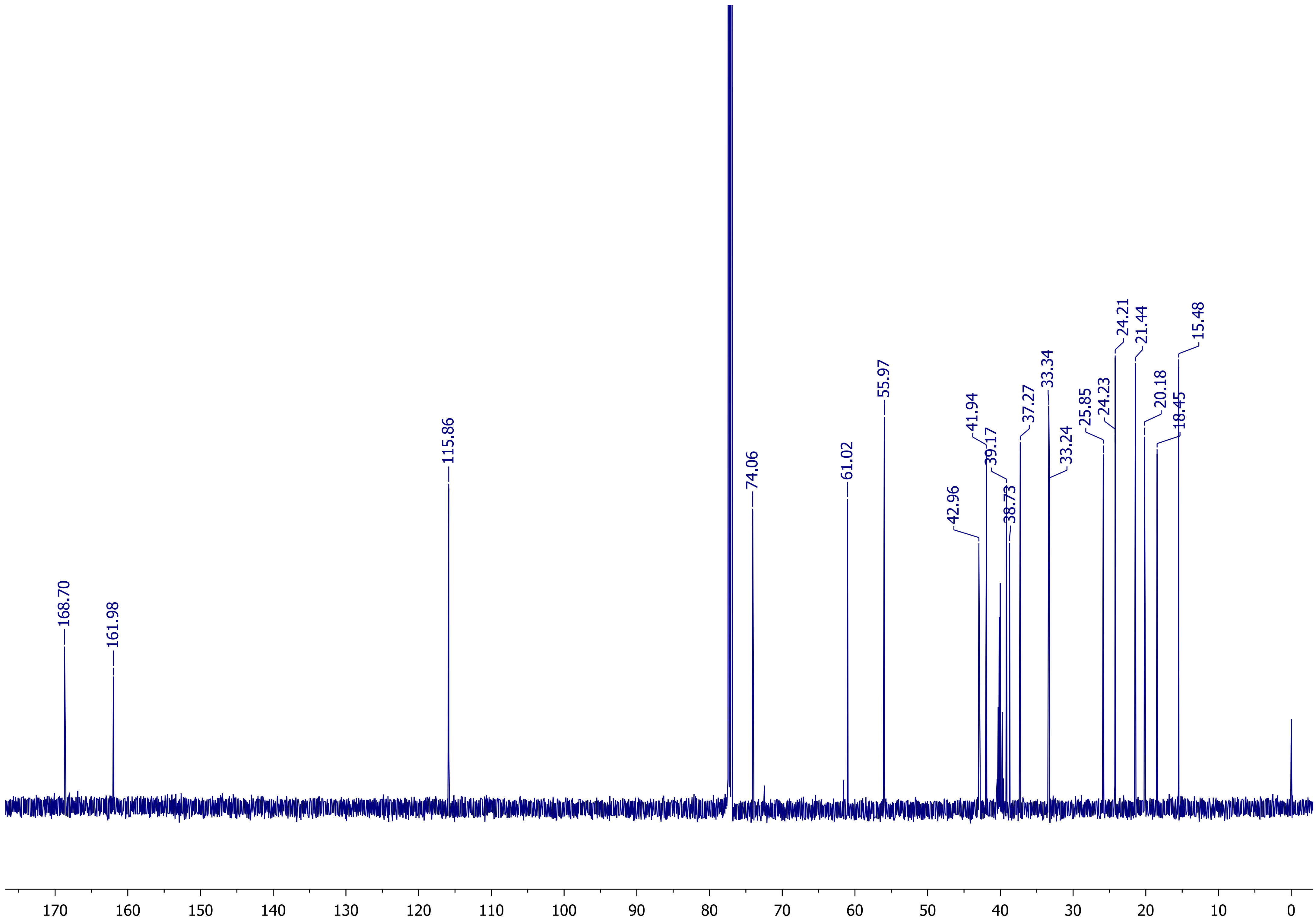

168.70
161.98
115.86
74.06
61.02
55.97
42.96
41.94
39.17
38.73
37.27
33.34
33.24
25.85
24.23
24.21
21.44
20.18
18.45
15.48
170
160
150
140
130
120
110
100
90
80
70
60
50
40
30
20
10
0

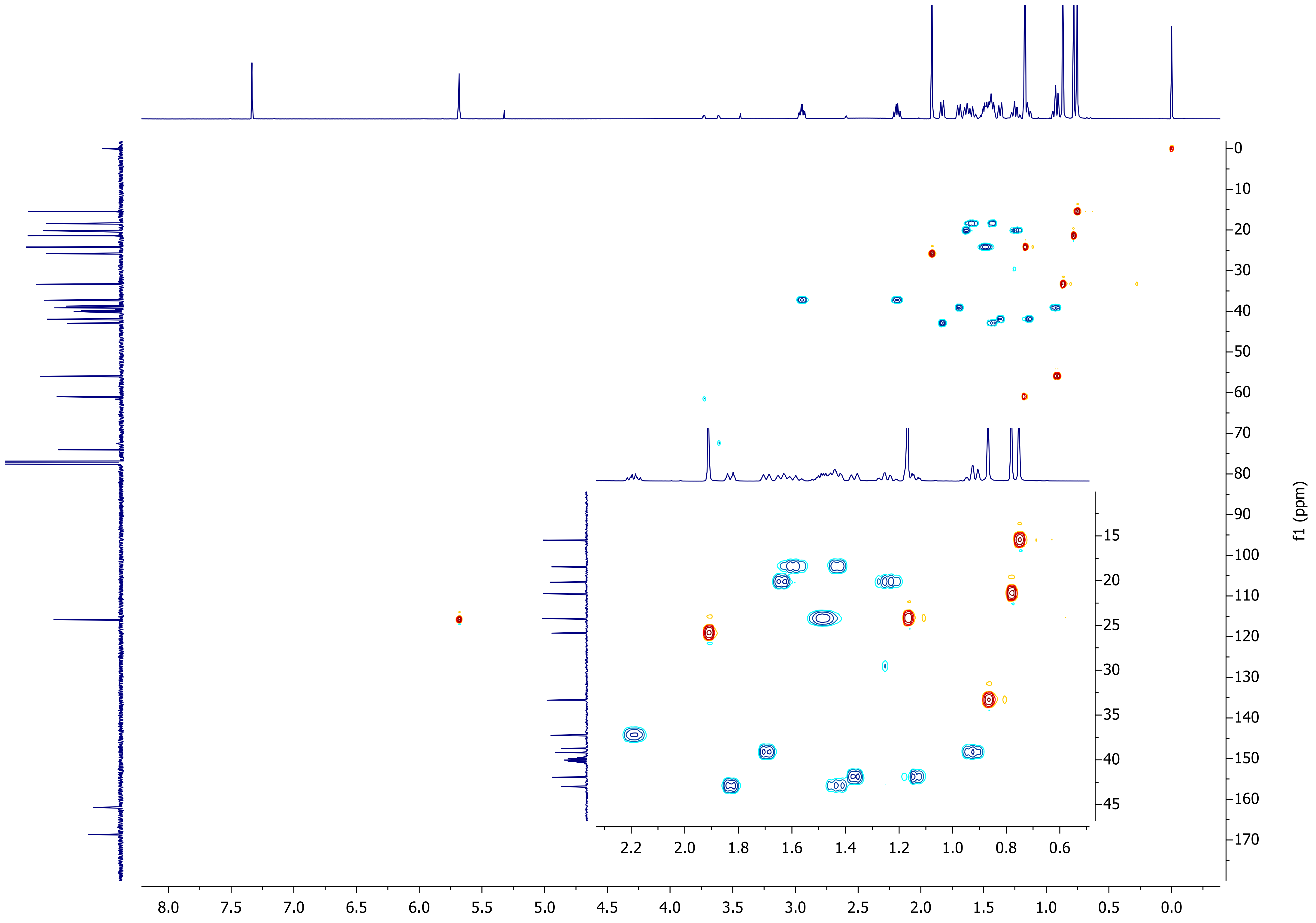

f1 (ppm)

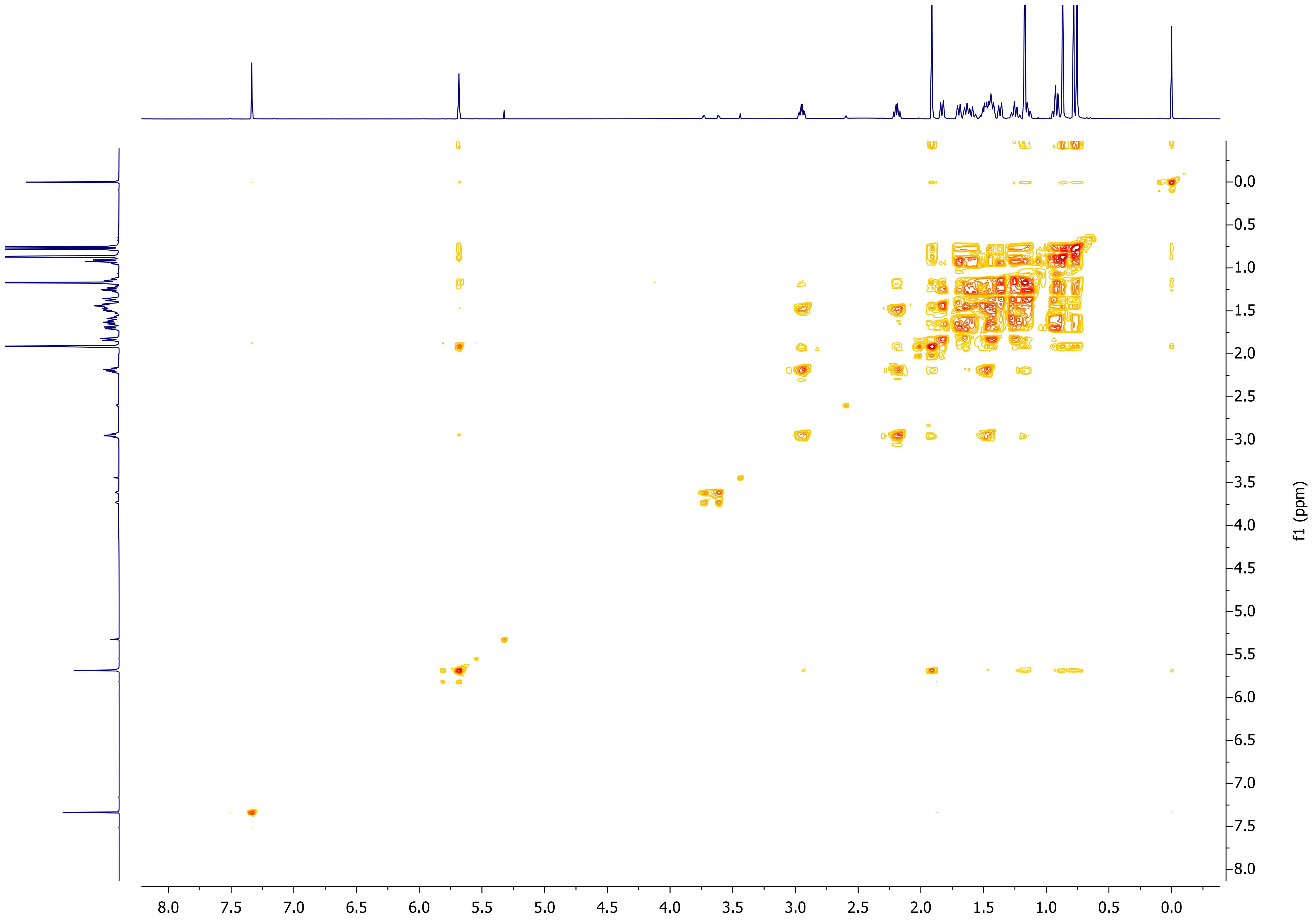

8.0
7.5
7.0
6.5
6.0
5.5
5.0
4.5
4.0
3.5
3.0
2.5
2.0
1.5
1.0
0.5
0.0
f1 (ppm)

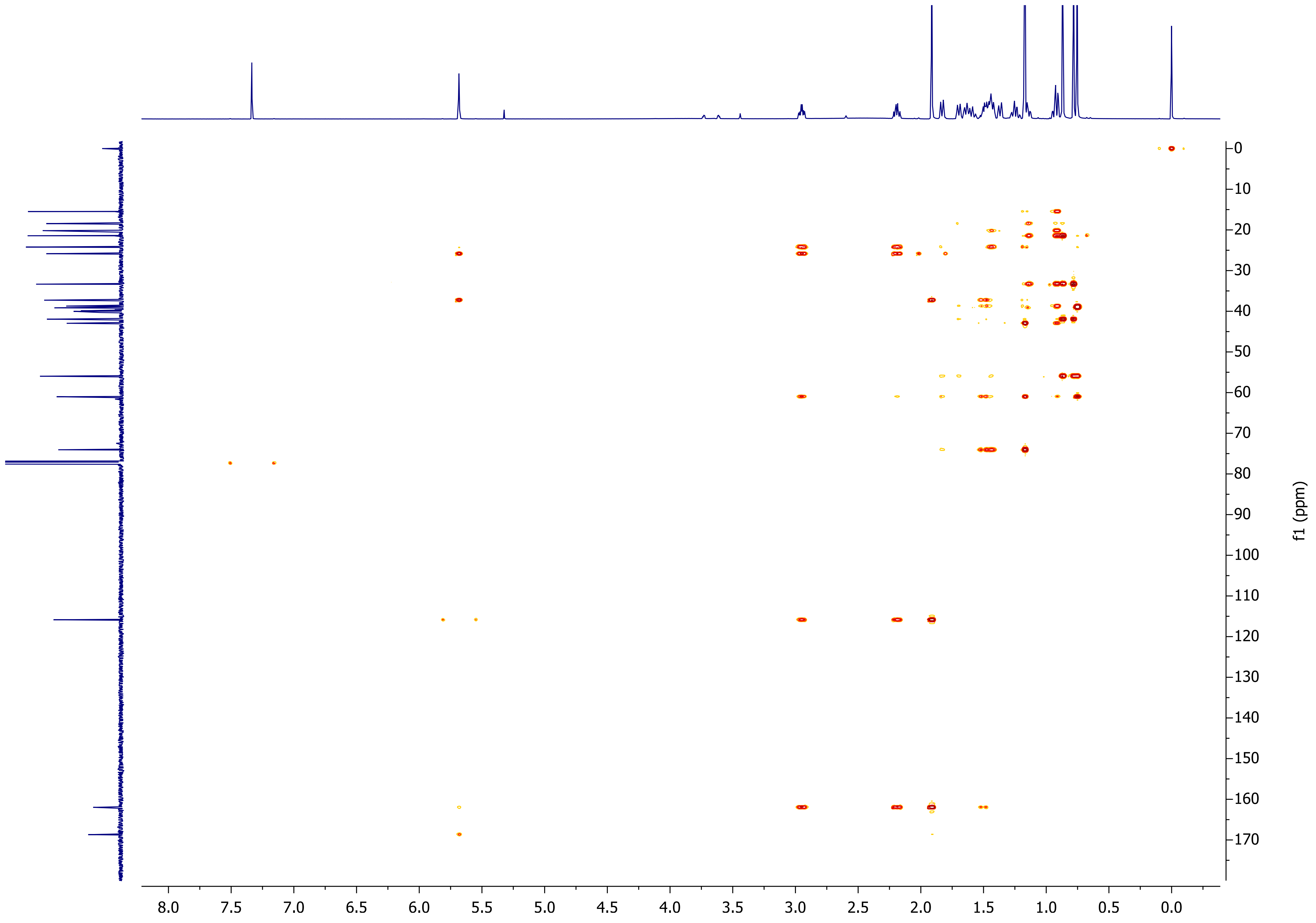

8.0
7.5
7.0
6.5
6.0
5.5
5.0
4.5
4.0
3.5
3.0
2.5
2.0
1.5
1.0
0.5
0.0
0
10
20
30
40
50
60
70
80
90
100
110
120
130
140
150
160
170
f1 (ppm)
